# Locating Mine Microseismic Events in a 3D Velocity Model through the Gaussian Beam Reverse-Time Migration Technique

**DOI:** 10.3390/s20092676

**Published:** 2020-05-08

**Authors:** Yi Wang, Xueyi Shang, Kang Peng

**Affiliations:** 1 School of Earth Sciences and Engineering, Sun Yat-sen University, Guangzhou 510275, China; ghost-zzz@163.com; 2 State Key Laboratory of Coal Mine Disaster Dynamics and Control, School of Resource and Safety Engineering, Chongqing University, Chongqing 400044, China; pengkang@cqu.edu.cn

**Keywords:** mine microseism, Gaussian beam, reverse-time migration location, 3D velocity

## Abstract

Microseismic (MS) source location is a fundamental and critical task in mine MS monitoring. The traditional ray tracing-based location method can be easily affected by many factors, such as multi-ray path effects, waveform focusing and defocusing of wavefield propagation, and low picking precision of seismic phase arrival. By contrast, the Gaussian beam reverse-time migration (GBRTM) location method can effectively and correctly model the influences of multi-path effects and wavefield focusing and defocusing in complex 3D media, and it takes advantages of the maximum energy focusing point as the source location with the autocorrelation imaging condition, which drastically reduces the requirements of signal-to-noise ratio (SNR) and picking accuracy of P-wave arrival. The Gaussian beam technique has been successfully applied in locating natural earthquake events and hydraulic fracturing-induced MS events in one-dimensional (1D) or simple two-dimensional (2D) velocity models. The novelty of this study is that we attempted to introduce the GBRTM technique into a mine MS event location application and considered utilizing a high-resolution tomographic 3D velocity model for wavefield back propagation. Firstly, in the synthetic test, the GBRTM location results using the correct 2D velocity model and different homogeneous velocity models are compared to show the importance of velocity model accuracy. Then, it was applied and verified by eight location premeasured blasting events. The synthetic results show that the spectrum characteristics of the recorded blasting waveforms are more complicated than those generated by the ideal Ricker wavelet, which provides a pragmatic way to evaluate the effectiveness and robustness of the MS event location method. The GBRTM location method does not need a highly accurate picking of phase arrival, just a simple detection criterion that the first arrival waveform can meet the windowing requirements of wavefield back propagation, which is beneficial for highly accurate and automatic MS event location. The GBRTM location accuracy using an appropriate 3D velocity model is much higher than that of using a homogeneous or 1D velocity model, emphasizing that a high-resolution velocity model is very critical to the GBRTM location method. The average location error of the GBRTM location method for the eight blasting events is just 17.0 m, which is better than that of the ray tracing method using the same 3D velocity model (26.2 m).

## 1. Introduction

As mineral resources exploitation goes deeper, the influence of dynamic disasters, such as fault slip, rockburst, and large area instability of rock mass becomes more and more serious [1,2,3]. This results in equipment damage, project delay, difficulties in recovering mineral resources, and threats to miner safety. Therefore, wideband and high-sensitivity sensors are equipped to record microseismic signals generated by these complex dynamic activities. By taking advantages of the mine microseismic (MS) signals, we can analyze characteristic parameters of these dynamic activities, such as event excitation time, source location, event magnitude, and focal mechanism. Based on these basic parameters, we can further infer the stress states of rock mass and take effective prevention and control. Among the above parameters, the MS event location can directly reflect location of dynamic activities, and it is the core foundations for calculating magnitude, inverting focal mechanisms, and evaluating risks of mine disasters [4,5,6]. The essence of the source location problem is to search the extreme values of a constructed objective functions defined by phase travel time residuals or waveform misfit, which can be considered as a standard nonlinear optimization problem. Up to now, a variety of MS event location methods have been proposed. According to differences in constructing objective functions (only using the travel time of specified seismic phase or the waveform information of finite band width), location methods can be generally classified into two categories, i.e., ray tracing-based location methods based on travel time and migration-based location methods using waveform processing.

### 1.1. Ray Tracing-Based Location Methods Based on Travel Time

The ray tracing-based location methods based on travel time are the most commonly used techniques for event location inversion, which unitizes the difference between the observed arrival time of specified phase and theoretical arrival time calculated by the ray tracing method in a given velocity model. The classical Geiger’s location method [7] is widely adopted in the inversion problem of seismology applications, which iteratively solves the linearized time difference equation. Some researchers have modified the type of objective function and iterative algorithm for the Geiger’s method to improve the convergence efficiency [8,9] based on arrival time of a single seismic phase. In order to better constrain source location, Zhou et al. [10] built an objective function combining the P-wave arrivals and travel time difference between P and S waves. Another widely used famous location method is the double difference approach proposed by Waldhauser and Ellsworth [11]. It is assumed that the propagation paths of wavefields generated by two adjacent seismic events are similar, which effectively reduces the influences of structural anomalies due to the similar ray path from receiver to adjacent earthquakes.

In spite of the objective function, the convergence of the inversion problem is also closely related to the selected optimization algorithm, thus many optimization algorithms have been applied to solve location problems. The Geiger’s location method adopts a first-order gradient descent algorithm, which is fast in computation but easily affected by the initial value. Thurber [9] and Li et al. [12] used the Newton and Gauss–Newton algorithm based on second-order Hessian to solve the inversion problem, improving the stability of the inversion but at the cost of longer computation time for calculating the second-order Hessian. Prugger and Gendzwill [13] and Li et al. [14] introduced the simplex method into the source location problem, obtaining a higher calculation speed and better location accuracy. Although the computation cost of using above algorithms is relatively small on the whole, they very easily fall into the problem of local minimum of the optimization. Therefore, some global search algorithms have been used in event location problem. Oye and Roth [15] determined MS event locations through a grid search way of the neighborhood algorithm in [16], which still calls for a large amount of computation. In addition, genetic algorithm [17,18], particle swarm algorithm [1], simulated annealing algorithm [19], and Bayesian algorithm [20,21,22] have also been introduced for source location and achieved a higher inversion efficiency and better location accuracy. Furthermore, the combination between grid search algorithm and global optimization algorithm is also a potential solution to improve the efficiency and constraints on location results. The conclusion is that ray tracing-based location methods based on travel time strongly depend on picking accuracy, which is the critical factor for the resolution of its location results.

### 1.2. Migration-Based Location Methods Using Waveform Processing

Compared with the ray tracing-based location methods only using travel time, the migration-based location methods utilizing waveform information make use of the windowed waveforms containing specified phase signal, which greatly reduce the requirement of seismic phase arrival picking precision and eliminate the influence of large picking errors that are caused by background noise [23,24] and MS signal misclassification for adjacent events [25]. In terms of migration forms and imaging conditions, the migration-based location methods using waveform processing can be classified as migration-based location methods using amplitude stacking idea, location methods based on seismic interferometry, and reverse-time migration location methods.

#### 1.2.1. Migration-Based Location Methods Using Amplitude Stacking

The amplitude stacking migration-based location method utilizes the Kirchhoff migration idea. All the recording waveforms are time-shifted and diffraction stacked to search the excitation time and source location. Kao and Shan [25,26] proposed the source scanning algorithm (SSA) based on absolute amplitude stacking of normalized waveforms, then Liao et al. [27] enhanced the brightness of the SSA method with an adaptive time window adjustment method. Gajewski et al. [28], Gharti et al. [29], and Grigoli et al. [30] separately carried out amplitude stacking migration-based location based on stacking of the square amplitudes, envelopes, and ratios of short time average to long time average (STA/LTA) of seismic waveforms. However, seismic waveforms received from different azimuths are closely related to event focal mechanism. Therefore, Liang et al. [31] and Yu et al. [32] put forward a joint source scanning algorithm considering source location and focal mechanisms, which increases the source location accuracy. Trojanowski and Eisner [33] systematically compared different amplitude stacking migration-based location methods and found that considering focal mechanisms and waveform polarization correction are important to improve source location performance. The amplitude stacking migration-based location method greatly reduces the requirements for specific phase arrival picking accuracy and can eliminate the influences of large picking errors through the waveform amplitude stacking procedure. In addition, it still resorts to traditional ray tracing to calculate travel time, which makes this method affected by multi-path effects as well as focusing and defocusing phenomena like ray tracing-based location method.

#### 1.2.2. Location Methods Based on Seismic Interferometry

Seismic interferometry location method takes advantage of the virtual waveforms generated by cross-correlation between different seismic waveforms, then imaging of the interferometric waveforms is used to determine the source location. The interferometric waveforms do not only retain the main characteristics of original waveforms but also reveal some stable characteristics that are difficult to be directly detected from original waveforms. Schuster et al. [34] discussed the calculation methods of seismic interferometry and its potential application fields, such as structural imaging and source location. Artman et al. [35], Witten and Shragge [36], and Wu et al. [37] determined source location by employing cross-correlation interferometry, convolution interferometry and deconvolution interferometry. Furthermore, other interferometry techniques, such as weighted-elastic-wave interferometric imaging [38], isotime point [39], and weighted deconvolution imaging [40] have been introduced to enhance location imaging quality of seismic interferometry. However, many conventional seismic interferometry location methods are still affected by the complexity of recording waveforms, inaccurate velocity models and sparse or uneven observation system.

#### 1.2.3. Reverse-Time Migration Location Methods

This method reconstructs underground wavefields through reverse-time extrapolation of wave equations, and the spatial location and excitation time of the MS event are obtained through a specific imaging condition. The basic operation steps are presented as follows: the windowed waveforms of specified seismic phases are taken as the input data; then, the back propagating wavefields are calculated by solving wave equations in reverse time. The source location and excitation time of an MS event are determined by taking the focusing point with the appropriate imaging condition. McMechan [41], Gajewski and Tessmer [42], and Larmat et al. [43] separately adopted a finite difference method and a spectral element method for wavefield back propagation and earthquake location. Li et al. [44] denoised MS signals with the shift-invariant dual-tree complex wavelet transform (DTCWT) and Birge–Massart threshold before reverse-time migration MS locating. By combining reverse-time location of wave equation extrapolation and interference imaging principle, Wang et al. [45] discussed a reverse-time location algorithm using interferometry of multi-source MS waveforms to improve accuracy and noise resistance of the reverse-time migration location method. While Zheng et al. [46] combined reverse-time imaging based on wave equations and travel time inversion in the frequency domain. Xue et al. [4] combined reverse-time migration with the least square iterative inversion to conduct reverse-time imaging of an MS source, thus iteratively improving the location accuracy. Furthermore, some researchers tried to improve the reverse-time migration imaging resolution by discussing and testing different imaging conditions. Nakata and Beroza [47] proposed a location algorithm called GmRTM by using the geometric mean as imaging conditions, which improves spatial resolution of source location. Sun et al. [48] and Zhu et al. [49] performed hybrid cross-correlation imaging condition by multiplication reduction on grouped back propagating wavefields from each receiver to compute a high-resolution microseismicity image. On this basis, Li et al. [50] employed a waveform inversion approach to obtain a finer resolution microseismic source location result to balance the trade-off between computation efficiency and location resolution. Xue et al. [51] incorporated shaping regularization imposing structure constraints on the estimated model into a reverse-time migration approach to attenuate migration artifacts and crosstalk noise, which has the potential of further improving the source location resolution. Song et al. [52] underlined the importance of reverse-time migration location in their subsurface camera (SAMERA) network idea, pointing out that interdisciplinary collaboration is the future direction for efficiently obtaining the in situ and real-time seismic inversion results based on advanced wireless sensor networks with distributed imaging algorithms.

In view of its advantages in locating events with low SNRs, the reverse-time migration location methods have been widely used in the fields of natural earthquakes [43], oil and gas exploitation-induced earthquakes [53], volcanic earthquakes [54,55,56], and glacial earthquakes [57]. The location method based on reverse-time migration of recording wavefields does not require specified phase arrival picking and is especially suitable for locating MS event with low SNR. However, this wave-equation-based technique requires a highly accurate velocity model, and the numerical solvers (e.g., finite difference method and spectral element method) have a huge computational cost.

### 1.3. Gaussian Beam Migration Technique

Forward wavefield modeling method based on a Gaussian beams approach, simultaneously using the ray tracing technique and numerical solver, is a compromise technique for effectively and accurately solving wave equations. It is a dynamic expansion of the approximate solution to wave equation through ray tracing method, and has encouraged migration imaging in the application of exploration geophysics, especially suitable for wavefield migration imaging under complex geological conditions. Hill [58] laid a theoretical foundation for the Gaussian beam migration technique, then a series of practical beam migration techniques have been derived, such as Li et al. [59], who proposed a beamforming technique-based simplified Gaussian beam construction to achieve sufficient accuracy and boost computation/communication efficiency. There are mainly two steps to realize Gaussian beam migration: using a single independent Gaussian beam for forward wavefield modeling along one direction and stacking Gaussian beams emitted from all directions for the final imaging. The Gaussian beam technique selects a series of appropriate ray parameters to simulate the wavefields based on Gaussian beam expansion in an independent central ray coordinate system, which does not need time-consuming two-point ray tracing (an example is shown in the Table 1 of Rawlinson and Sambridge [60]). It can effectively model the focusing, defocusing, diffraction, and multi-path effects. Furthermore, Gaussian beam migration has integrated the flexibility of Kirchhoff-type (diffraction stacking) migration and high precision of wavefield extrapolation migration. In conclusion, the Gaussian beam modeling and corresponding migration technique is a delicate, accurate, flexible, and efficient simulation and imaging approach.

According to these unique advantages of the Gaussian beam migration technique, some researchers have introduced it into studies of locating natural earthquakes [61] and hydraulic fracturing-induced earthquakes [59,62,63]. In the above applications, 1D layered, 2D, and/or very simple 3D velocity models are used to locate the source, which proves the robustness of the reverse-time migration location technique based on Gaussian beams and their potential in detecting MS events. However, to the author’s best knowledge, this technique has not been applied in complex 3D inhomogeneous media of mining regions. In addition, surface observation systems or evenly spaced vertical sensors in wells are used in the Gaussian beam reverse-time MS event location. In this study, the sensors were arranged in the irregular underground mine tunnels, where the velocity structure presented strong 3D heterogeneity. Therefore, it is necessary to modify and re-test the algorithms, and write programs that are suitable to the 3D velocity model and mine observation system to enhance the applicability of Gaussian beam migration technique in locating mine MS events.

### 1.4. What Will be Done in This Work

The contributions and innovations of this article are as follows:

(1) We introduced the GBRTM technique into locating a mine microseismic event and considered a tomographic complex 3D velocity model. The GBRTM location images for realistic data application emphasized that the quality and resolution of the location results is dominantly controlled by the accuracy of 3D velocity model.
(2) We used irregular underground networks instead of surface or borehole dense sensor networks for the wavefield back propagation migration, and investigated the validity of GBRTM technique for stacking complicated and incoherent recording waveforms.

The rest of the contents are arranged as follows: Section 2 briefly introduces the GBRTM location method in a 3D velocity model. Then, the effectiveness of the GBRTM location method was tested by two synthetic tests in Section 3: the synthetic waveforms generated from the Ricker wavelet and the synthesized realistic monitored waveforms from blasting events. Meanwhile, the GBRTM location method using a 3D velocity model was applied to locate eight blasting events with premeasured locations, and the location results were compared with previous studies. The influences of arrival time and velocity model accuracy on the GBRTM location results are discussed in Section 4. Section 5 presents summary and prospects of this study.

## 2. Methodology

The Gaussian beam technique locally solves wave equations in the complex media, which simulates dynamic information such as wavefield amplitude and takes wavefront curvature variation of wavefield in inhomogeneous media into account. The wavefield forward modeling by Gaussian beam technique can be implemented from two steps, i.e., kinematic and dynamic ray tracing and Gaussian beam wavefield stacking. The former calculates a single independent Gaussian beam, while the latter makes clear that the Green’s function of wavefield at any spatial point can be obtained through a linear stacking of different outgoing Gaussian beams with effective contribution to the target point in its own neighborhood.

**Figure 1:**
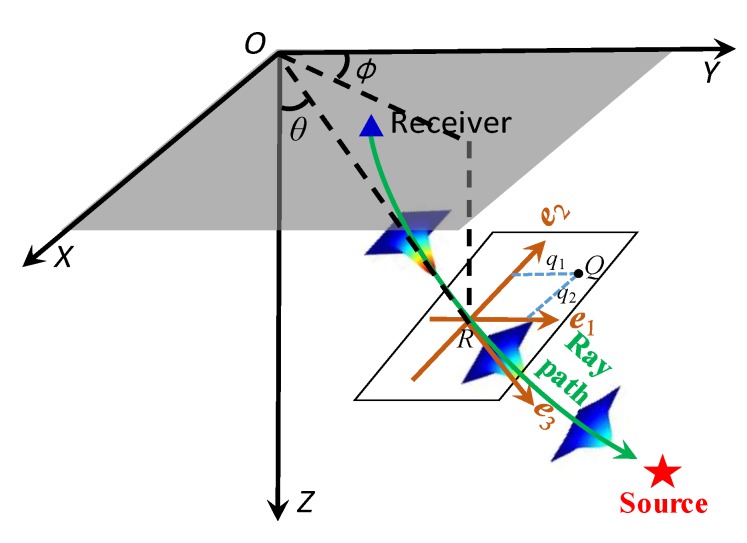
Central ray coordinate system of the 3D Gaussian beam wavefield modeling.

The Gaussian beam technique selects a series of appropriate ray parameters to simulate the wavefields based on Gaussian beam expansion in an independent central ray coordinate system. The central ray coordinate system of a 3D Gaussian beam is shown in Figure 1. It shows that the energy tubes form a Gaussian beam along the central ray, and the energy distribution of the beam attenuates along the distance deviating from central ray in the form of a Gaussian function. ***e***_1_, ***e***_2_, and ***e***_3_ represent the basic vectors of the central ray coordinate system (*q*_1_, *q*_2_, *s*) at point *R*. Note that ***e***_3_ indicates the tangential vector along the central ray, ***e***_1_ and ***e***_2_ denote two orthogonal normal vectors perpendicular to ***e***_3_. ***e***_1_, ***e***_2_, and ***e***_3_ can be expressed in global coordinate system as follows:

(1) $$\left\{ \begin{array}{l} e_{1}=(\cos\theta \cos\phi ,\cos\theta \sin\phi ,-\sin\theta )\\ e_{2}=(-\sin\phi ,\cos\phi ,0)\\ e_{3}=(\sin\theta \cos\phi ,\sin\theta \sin\phi ,\cos\theta ) \end{array}\right.$$

where θ indicates the angle between ***e***_3_ and vertical direction, ϕ represents the azimuth angle of tangential vector ***e***_3_ at a point on the central ray system.

Cerveny et al. [64] gave the solution of wavefields using the 3D Gaussian beams approximation at point *Q* in the central ray coordinate system.

(2) $$U_{\mathrm{GB}}\left(s,q_{1},q_{2},\omega \right)=\sqrt{\frac{V(s)\det\left[Q(s_{0})\right]}{V(s_{0})\det\left[Q(s)\right]}}\exp\left[i\omega \tau +\frac{i\omega }{2}q^{T}M(s)q\right]$$

where $V(s_{0})$ represents the initial velocity of the central ray at the source point; $q^{T}=\left(q_{1},q_{2}\right)$, in which $q_{1}$ and $q_{2}$ indicate the coordinates of point *Q* along the local coordinate axes ***e***_1_ and ***e***_2_; *s* denotes the length of the ray path calculated from the source along the central ray path, ω is the frequency of wavefields for Gaussian beam modeling. Matrix M(s)=P(s)/Q(s) where ***Q***(*s*) and ***P***(*s*) are dynamic parameters matrixes of the Gaussian beam; the real part of M(s) characterizes wavefront curvature of the Gaussian beam, while the imaginary part determines the attenuation characteristics of transverse amplitudes perpendicular to the direction of tangent vector along the ray ***e***_3_. ***Q***(*s*) and ***P***(*s*) obey the first-order ordinary differential equation system:

(3) $$\left\{ \begin{array}{l} \frac{dQ(s)}{d\tau }=V^{2}(s)P(s)\\ \frac{dP(s)}{d\tau }=-\frac{1}{V(s)}V(s)Q(s) \end{array}\right.$$

where ***V***(*s*) indicates the velocity field at the local point *Q*; $V_{ij}\left(s\right)=\frac{\partial^{2}V(s)}{\partial q_{i}\partial q_{j}}$ (*i*, *j* = 1, 2), representing the matrix of second-order partial derivatives of velocity field at the point *Q* in the central ray coordinate system along the local transverse ***e***_1_ and ***e***_2_ directions.

In a 3D isotropic media, assuming that $x_{s}=\left(x_{s},y_{s},z_{s}\right)$ and $x_{r}=\left(x_{r},y_{r},z_{r}\right)$ are coordinates of the source and receiver, the 3D Green’s function by wavefield back extrapolation from the receiver $x_{r}=\left(x_{r},y_{r},z_{r}\right)$ to any spatial point x can be constructed by stacking integral of Gaussian beams with different ray parameters [58].

(4) $$G(x,x_{r},\omega )=\frac{i\omega }{2\pi }\iint \frac{dp_{x}dp_{y}}{p_{z}}U_{GB}(x,x_{r},p,\omega )$$

where $p_{x}=\frac{\sin\theta \cos\phi }{V(s_{0})}$, $p_{y}=\frac{\sin\theta \sin\phi }{V(s_{0})}$ and $p_{z}=\frac{\cos\theta }{V(s_{0})}$ are initial ray parameters at receiver and indicate the take-off angle of emitting Gaussian beams. The interval of initial ray parameters should be small enough in order to ensure that the Green’s function stacked by Gaussian beams based on integral Equation (4) physically present a point-source wavefield with a finite bandwidth and avoiding aliasing issues. This requirement means that the variation of travel time of two adjacent Gaussian beams from receiver to spatial point has to be less than half of dominant period. If the integral Equation (4) is discretized, the interval of the initial ray parameters should be related to the frequency bandwidth of seismic waveforms with the value usually set as the reciprocal of the product between the highest cut-off frequency of wavefield and initial width of Gaussian beam. More details of the choice for initial parameters for Gaussian beam modeling, such as initial beam width and modified weighting factor for beam stacking, can be found in [64].

If the finite fracturing process of a seismic source is not considered, the MS and blasting sources in the mining zone can usually be simplified as a point source. The theoretical waveform $u_{i}\left(x,x_{s},\omega \right)$ at the point x in the *i* direction can be obtained by the convolution between the partial derivative of the Green’s function $G_{ij,k}(x,x_{r},\omega )$ and the point source moment tensor $M_{jk}(x_{s})$, that is,

(5) $$u_{i}\left(x,x_{s},\omega \right)=\sum_{j=1}^{3}\sum_{k=1}^{3}G_{ij,k}(x,x_{r},\omega )\times M_{jk}(x_{s}).$$

This study only exploits the recording Z-component windowed waveforms for reverse-time back propagation due to the fact that single-component sensors are mainly used in a mine MS monitoring system. The value of energy focusing at the target point x after back propagation wavefield stacking from all sensors is:

(6) $$I(x)=\sum_{l=1}^{L}u_{zl}\left(x,x_{s},\omega \right)$$

where L represents the number of waveforms data for wavefield back propagation; $u_{zl}\left(x,x_{s},\omega \right)$ indicates the Z-component windowed extrapolation waveform through reverse-time back propagation from the *l*th sensor to target point x. Finally, through Parseval’s theorem and corresponding integral operation in the frequency domain of the stacking Equation (6), we define a focusing imaging condition at each target point x by collapsing the time axis to extract the zero-lag of autocorrelation of stacking waveforms in physical space x [35]. All sensors in the acquisition system contain the MS event and thus have measurable autocorrelation value. The energy accumulation leads to high amplitude value at the event location based on autocorrelation operation. Another important advantage of the autocorrelation imaging condition is that the squared energy imaging condition is more stable than the case of complicated stacking wavefields. Squaring penalizes small values that are likely crosstalk and artefacts. In summary, the GBRTM location method in a 3D velocity model is realized in the following steps: firstly, the MS signals are selected and appropriately filtered; then, waveforms containing P-wave arrival are windowed by the simple tapering operation shown in the study of Wang et al. [65]; next, the target area is divided into small volume elements according to the resolution requirements of location accuracy, and the wavefield of a single independent Gaussian beam at each point in the space can be modeled based on a certain azimuth and initial ray parameters; after spatially stacking and squaring the back extrapolation wavefields of all effective Gaussian beams from all sensors, the point with the maximum focusing energy is considered as the source location.

## 3. Results

### 3.1. Synthetic Test

#### 3.1.1. Synthetic Dataset

**Figure 2:**
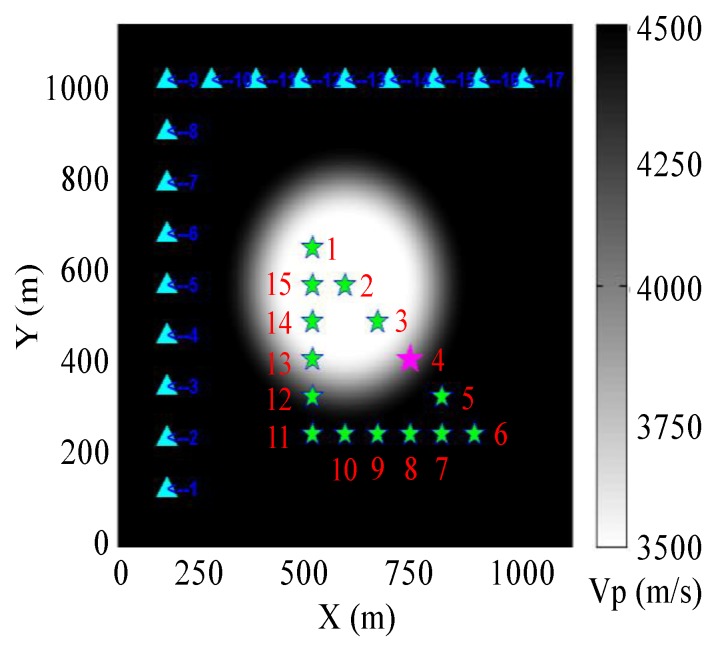
Synthetic test model for the Gaussian beam reverse-time migration (GBRTM) location method. The grey colorbar shows the velocity value of the test model. Here, only the 2D model with a low-velocity anomaly is illustrated, and the model with a high-velocity anomaly has the same structural shape, and the only difference is that the low-velocity anomaly (3500 m/s) is replaced by the high-velocity anomaly (5500 m/s); Cyan triangles and green stars separately indicate locations of sensors and sources. The red numbers correspond to the id of the sensors and test events.

As a representative test of the GBRTM location method used in this research, a 2D velocity model (Figure 2) with a regular circular velocity anomaly is utilized to verify the location method. The 2D velocity model has dimensions of 1100 m × 1100 m. Since the velocity range of the target mine is about 3500–5500 m/s [66], a circular velocity anomaly is placed in the center with a background velocity 4500 m/s. Two sets of different velocity anomaly are set to be 3500 m/s (low-velocity anomaly) and 5500 m/s (high-velocity anomaly) as two synthetic tests. In order to reduce the complexities caused by wavefield reflection and scattering at sharp velocity boundary, the boundary of the velocity anomaly is smoothed. In the background velocity region, 17 sensors are arranged with a spacing of 110 m, and 15 test events are arranged in both high-velocity and low-velocity anomaly areas, with a distance of 80 m for adjacent events. The event source mechanism is set as an isotropic explosion, and the Ricker wavelet with a main frequency of 200 Hz is utilized to generate the source–time function to excite the wavefield. The synthetic waveforms are generated by the Ricker source wavelet or synthesized from the windowed real recorded blasting waveforms with modeled source-to-receiver travel time shifted from the synthetic model. The reason why two sets of synthetic data are used for test is that the commonly used Ricker source wavelet had very good spectrum distribution characteristics and analytical properties. Only the influence of geometric spreading caused by structural change is considered in the synthetic test using the Ricker wavelet and, thus, the generated waveforms are relatively simple, while the spectrum characteristics of realistic recoded waveforms are far more complex, which can better demonstrate the robustness and effectiveness of the GBRTM location method by including them into synthetic tests.

#### 3.1.2. Synthetic Test Examples

##### Test Example of the Ricker Source Wavelet

**Figure 3:**
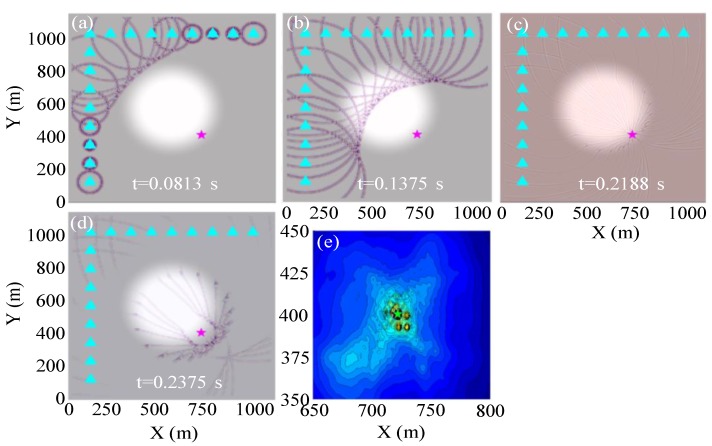
Location results of the GBRTM location method when using the Ricker wavelet with a dominant frequency of 200 Hz for synthetic test. (**a**–**d**) Stacking back propagation wavefields from each sensor at different time based on Gaussian beam modeling. The background is the 2D velocity model and the pink star indicates the true location of the test source. (**e**) Stacking amplitude imaging of back propagation wavefields at points near the true source based on autocorrelation imaging condition. The green star shows the source location determined by the GBRTM location method, which almost covers the true source location represented by a pink star.

Event 4 (pink star in Figure 2) at the boundary of the low-velocity anomaly is taken as an example. The stacking back propagation wavefields of each sensor at different time and amplitude focusing imaging near the true source location are displayed in Figure 3 when conducting the GBRTM location test based on synthetic data from the Ricker wavelet with 200 Hz dominant frequency. It can be seen that with the increasing propagation distance of Gaussian beams, the wavefields quickly attenuate due to the effects of geometric spreading (approximately inversely proportional to the propagation distance). Moreover, the wavefields propagate faster in the high-velocity zones. However, the delayed wavefront disturbance caused by the low-velocity anomaly gradually reduces as the propagation distance increases (Figure 3c,d), that is, the finite frequency wavefront healing phenomenon [67,68]. The reverse-time propagation of wavefields does not need to consider the picking of seismic phase arrival and scanning excitation time. The excitation time of MS event can be automatically obtained by the maximum time value of the stacking wavefield time series of the fully focusing point (at the time shown in Figure 3c). This focusing point is determined from maximum spatial energy of stacking wavefields during the back propagation. Furthermore, compared with high-frequency 3D ray propagation modeling [66], the changes of wavefront curvature and amplitude of back propagation wavefields based on Gaussian beam modeling can be described by the changes of the dynamic parameters matrix in Equation (2), which effectively and reasonably simulates focusing, defocusing, and multi-path effects. Energy focusing imaging at points near the true source using the GBRTM technique and autocorrelation imaging condition is shown in Figure 3e. The imaging results show a good focusing effect, and the distance between the location of preset true source and the location result is less than 2 m. In addition, for the remaining 14 events and the 15 events using the 2D model with the high-velocity anomaly, the location errors are all smaller than 2 m, which verifies the effectiveness of the location method.

##### Test Example of Synthesized Realistic Blasting Waveforms

**Figure 4:**
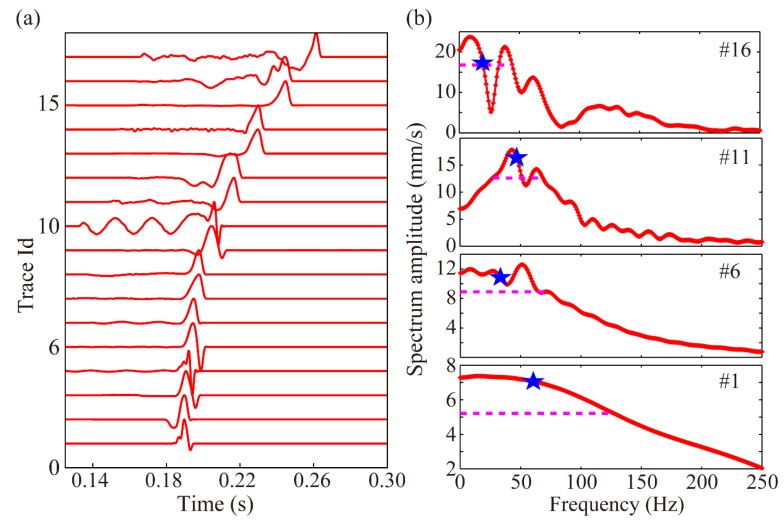
Windowed waveforms of the realistic blasting event 1 (**a**) and representative amplitude spectra of some windowed waveforms (**b**). The blue star represents the main frequency of the waveform and the pink dashed line corresponds to the approximate dominant frequency band.

**Figure 5:**
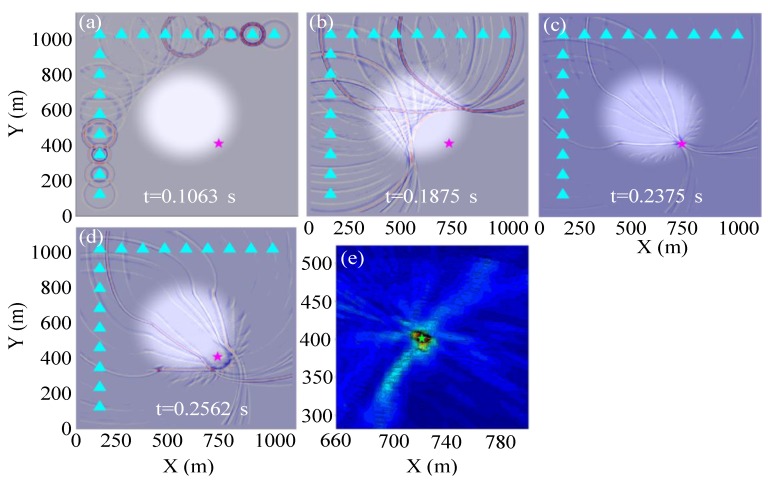
Location results of the GBRTM location method when using synthetic waveforms from the realistic blasting event 1. (**a**) t = 0.1063 s; (**b**) t = 0.1875 s; (**c**) t = 0.2375 s; (**d**) t = 0.2562 s; (**e**) Stacking amplitude imaging of back propagation wavefields at points near the true source based on autocorrelation imaging condition. The rest of the instructions are the same as in Figure 3.

Event 4 is used for illustration again, and the GBRTM location method is tested using synthesized waveforms based on the real recording blasting event 1 introduced in Section 4. The windowed realistic blasting waveforms containing the first dominant period of direct P-wave are shown in Figure 4a, and the amplitude spectra of some typical windowed waveforms are illustrated in Figure 4b. Here, the blasting waveforms are shifted according to the source-to-receiver travel time based on the Gaussian beam wavefield modeling in the 2D velocity model. It can be seen that the windowed blasting waveform is far more complex and has a wider approximate dominant frequency band distribution [61] and obviously different main frequency property compared with the simple Ricker source wavelets, where the approximate dominant frequency band is defined as the frequency range between the first and last frequency points at 0.707 times of the maximum spectrum amplitude; when there is just one frequency point that satisfies the above spectrum amplitude range setting, the first frequency point is set as 0 Hz. Therefore, this synthetic test is more similar to a real MS event location application. The stacking back propagation wavefields from each sensor at different times and final amplitudes focusing imaging near the true source location are illustrated in Figure 5. The back propagation wavefields are more complicated than those from the Ricker wavelet. The expansion ranges of back propagation wavefront from different sensors differ significantly due to different level of noises and length of wave train shapes shown in Figure 5a. In any case, the imaging results show that the GBRTM location method still obtains a stable location focusing capability with an error of 1.7 m. We conducted a similar synthetic test but using waveforms synthesized from the realistic blasting event 3, which has a higher level noise (Figure S1). However, a good imaging result with location error of 1.9 m is obtained, as shown in Figure S2. The remaining synthesized blasting event waveforms and the model with high-velocity anomaly are also tested with location errors all smaller than 3 m (Table 1), demonstrating that the GBRTM technique is robust and feasible in an MS event location.

#### 3.1.3. Synthetic Results Based on Different Velocity Models

**Figure 6:**
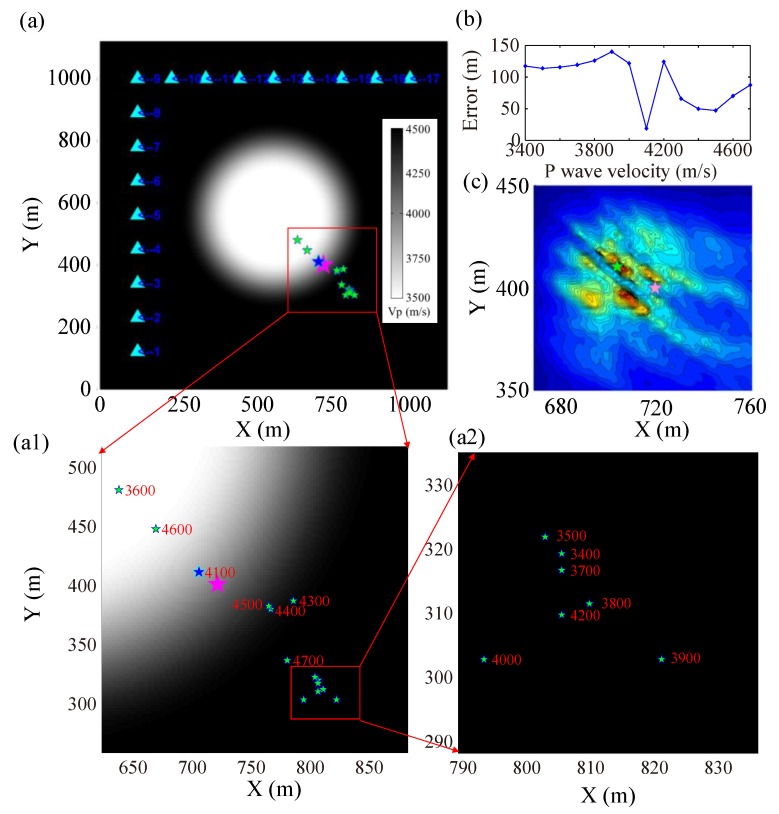
Location results of the test source 4 by using the GBRTM location method for different homogeneous velocity models. (**a**) Location results obtained by the GBRTM location method with different homogeneous velocity models. The pink star denotes the true source location and the small green stars represent the locations determined by the GBRTM method with homogeneous velocity models increasing from 3400 m/s to 4700 m/s. The blue star indicates source location determined by the GBRTM method with the minimum location error under a homogeneous velocity model with the value of 4100 m/s. (**a1**,**a2**) are enlarged subplots of the (**a**), and the numbers correspond to the used homogeneous velocity model. (**b**) Location errors of the GBRTM method using different homogeneous velocity models. (**c**) Stacking amplitude imaging at points near the true source location based on autocorrelation imaging condition when the background velocity is 4100 m/s. The green star and the pink star indicate the location obtained by the GBRTM method and true source location.

In order to better illustrate the effect of velocity model accuracy for the GBRTM location method, the synthesized waveforms generated by 2D test model combined with different homogeneous velocity models for migration are treated as comparisons. The test source 4 is again taken as an example, and the location results of the GBRTM technique with different homogeneous velocity models are shown in Figure 6, in which the P-wave velocity of homogeneous velocity models used here increases from 3400 to 4700 m/s at a rate of 100 m/s, it can be seen that location errors of the GBRTM technique are mostly 50–150 m, and the minimum location error 18.81 m is achieved when the background P-wave velocity is 4100 m/s. However, the stacking amplitude imaging of back propagation wavefields in homogeneous velocity model of 4100 m/s is very divergent at points near the true source (Figure 6c), so the location result is unstable.

**Figure 7:**
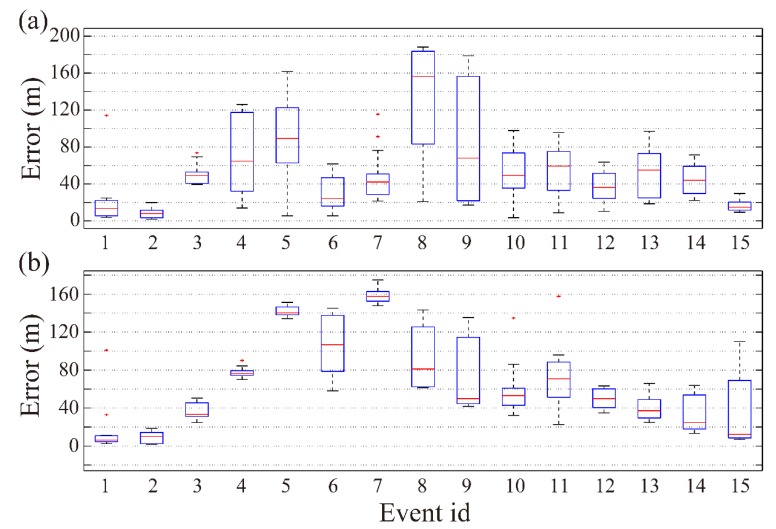
Location error boxplots of the 15 test events using the GBRTM location method under different homogeneous velocity models (from 3400 to 4700 m/s). (**a**) Boxplots of location errors under the synthetic data from 2D velocity model with the low-velocity anomaly; (**b**) Boxplots of location errors under the synthetic data from 2D velocity model with the high-velocity anomaly.

The boxplot of location results for all the 15 events in different homogeneous background velocity models are presented in Figure 7, while location errors of the GBRTM technique using the correct 2D velocity model of Figure 2 are demonstrated in Table 1. The location errors of the GBRTM technique based on the correct 2D velocity model are all smaller than 3 m, while most location errors of the GBRTM technique utilizing homogeneous velocity models are large, ranging from 20 to 150 m. The test events 1, 2, and 15 with location errors smaller than 20 m have small average distances from sources to sensors, and therefore, the wavefields of these events are less affected by the preset velocity anomalies and the variations of heterogeneous velocity structure have relatively small influences on corresponding wavefield propagation. In addition, the coverage of the sensor array used in the synthetic test has also better constraints on source locations for these 3 test events.

The synthetic tests on the GBRTM location method under different velocity models illustrate that the GBRTM method using simplified homogeneous velocity model exhibits significant location errors; in other words, the accuracy of the velocity model plays a critical role in reverse-time migration location method based on waveform back propagation modeling. Due to the complexity of practical velocity model in a mining zone, it is necessary to consider the combination of a high-resolution 3D velocity model and the waveform-based GBRTM location method.

### 3.2. Application Test

#### 3.2.1. Application Dataset

**Figure 8:**
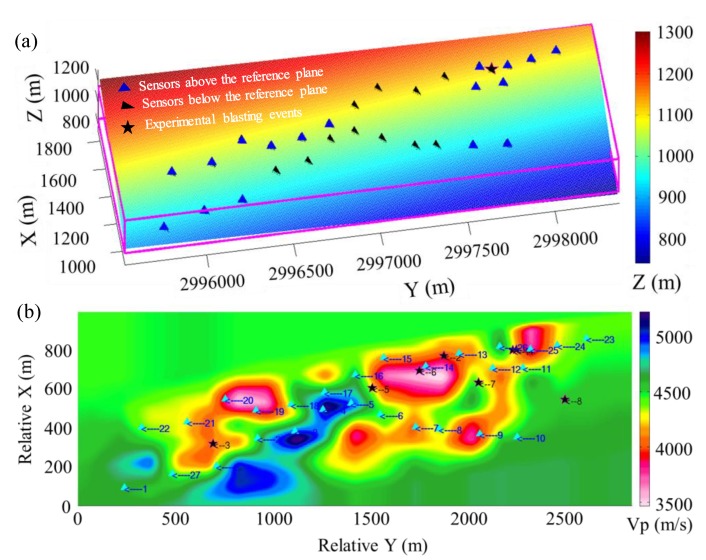
3D spatial location of the 2D slice in tomographic velocity model and 28 sensors and experiment blasting events in the Yongshaba mine. (**a**) The 2D velocity slice is defined by the plane with the smallest projection distance to all sensors, and the origin of the relative plane coordinates corresponds to the coordinate point in the lower left corner. The positive Z direction is vertically upward, while the X and Y directions are respectively along the north and east. Colorbar represents the elevation of the 2D slice in 3D coordinates. The blue triangle represents those locations of sensors that are actually above the 2D slice, while the black triangle indicates the locations of a sensors that are below the slice. Black stars are the projection locations of the eight experiment blasting events onto the 2D slice. (**b**) The 2D velocity model view corresponding to the 2D spatial slice. It is obtained through interpolation of the high-resolution 3D P-wave velocity model of Wang et al. [65]. The numbers beside the cyan color triangles and the black stars correspond to the index of the sensors and the blasting events projected onto 2D slice.

The Yongshaba mine, located in Kaiyang County, Guizhou Province (China), has more than 20 main faults and a lot of cavities left after mining, which may induce geological disasters such as fault slip and a large area of rock mass failure [69]. Therefore, a set of Institute of Mine Seismology (IMS) monitoring systems including 28 sensors with a sampling frequency of 6000 Hz was arranged, in which 12 sensors were separately arranged on layers of 930 and 1080 m and four sensors were placed on the layer at 1120 m (Figure 8). To verify the practical feasibility of the GBRTM location method with the high-resolution 3D tomographic velocity model inverted by Wang et al. [65], eight blasting events with premeasured locations were taken for the realistic test. The number, excitation time, and true location of the eight blasting events as well as the number of triggered sensors are listed in Table 1 of Wang et al. [66].

For better illustration, a 2D velocity spatial slice is selected to show the imaging results from GBRTM location method and back propagation reverse-time wavefields based on Gaussian beam modeling. The 2D velocity slice is defined by the plane with the smallest projection distance to all sensors, and the origin of the relative plane coordinates corresponds to the coordinate point in the lower left corner illustrated in Figure 8a. According to distributed locations of the sensors and complexity of velocity model shown in Figure 8b, it can be seen that P-wave velocity in the mining area of the Yongshaba mine shows very strong heterogeneity due to the effects of mining activities (e.g., ore excavations and blasts) and complex geological conditions (Figure 15 in [65]). Figure 15 in [65] shows that the low-velocity (low-V) zone and high-velocity (high-V) zones match well with the geological setting and the excavation plan: the low-V zone may reflect empty volumes, stress release events, and rock breaking and cracking caused by mining activities, while the high-V zones are probably the consequences of local stress concentrations caused by regional stress redistribution [65].

The maximum velocity perturbation can reach 2000 m/s, and there are typical high-velocity and low-velocity regions. As a result, obvious multi-path effects and focusing and defocusing phenomena exist, which will enhance the nonlinear characteristics of inversion based on the traditional ray tracing method. In addition, due to frequent mining activities and limitations of field conditions in mining zones, the SNR of MS recording waveform is relatively low. Moreover, the ray tracing-based location method using arrival time strongly depends on the picking accuracy of the specified phase’s first arrival, which further limits its applicability. Considering these difficulties, the source location utilizing the GBRTM technique with the high-resolution tomographic 3D velocity model should be carried out to improve the location accuracy.

#### 3.2.2. Location Examples

**Figure 9:**
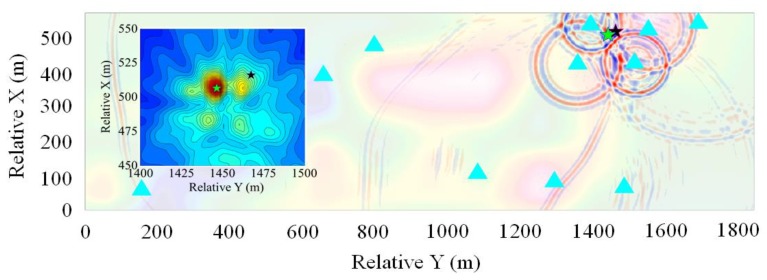
Location results of the GBRTM location method based on windowed recording waveforms of the blasting event 1. The 2D velocity slice shown in the transparency base map show is defined by the 2D spatial plane with the smallest projection distance to all sensors recording this event and containing the true source of blasting event 1. The time of wavefield plot correspond the moment when the wavefields from all the sensors are approximately focused at a point. The black star represents the actual location of the blasting event, while the green star represents the location determined by the GBRTM method. The left panel shows the enlarged map of the stacking amplitude imaging near the true source location using autocorrelation imaging condition.

**Figure 10:**
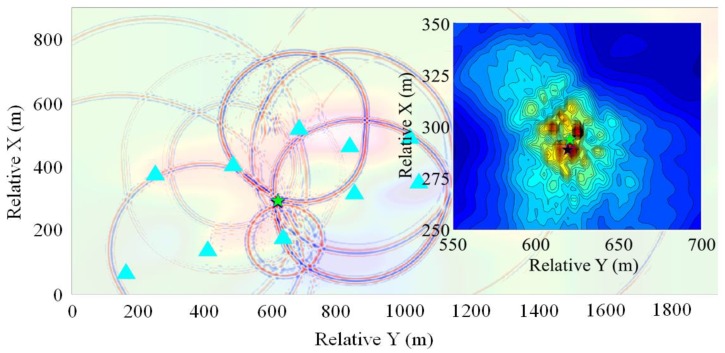
Location results of the GBRTM location method based on the windowed recording waveforms of the blasting event 3. The right panel shows the enlarged map of the stacking amplitude imaging near the true source location. The remaining legends are the same as those in Figure 9.

The location results of the blasting events 1 and 3 obtained by the GBRTM method are selected for illustration, and corresponding stacking back propagation wavefields from each sensor and stacking amplitude images obtained by the GBRTM method at points near the true sources are demonstrated in Figure 9 and Figure 10. The 2D velocity slice shown in the transparency base map is defined by the 2D spatial plane with the smallest projection distance to all sensors recording this event and containing the true source of blasting event. As shown in the figures, there is some noise in the back propagation wavefields through Gaussian beam modeling, which are caused by the scattering that occurred when wavefields encounter heterogeneous structures. When the wavefields propagate over a long distance, the wavefonts subjected to reverse-time back propagation from sensors will be affected by the cumulative effects of heterogeneous velocity structures, thus showing distinct irregular shape. The time of wavefield shown in the figure is the moment when the wavefields from all the sensors are appropriately focused at a point (namely, the location of focusing point). However, a few back propagation wavefields modeled by Gaussian beam technique is a bit far away from the energy focusing point. This may be due to inaccurate signal classification of different events and insufficient accuracy of velocity models resulting in a poor migration stacking result. Moreover, in addition to the initial P-wave arrival, other complex wave phenomena caused by structural anomalies in the windowed waveforms for migration may also degrade the stacking quality. Stacking imaging of the back propagation wavefields by Gaussian beam modeling for event 1 has a better focusing quality, while that of event 3 shows a certain degree of noises, leading to a slightly greater dispersion for the stacking amplitude imaging. Nevertheless, the regions with the maximum energy focusing of back propagation wavefields of both events are very close to the premeasured locations of the blasting events, indicating effectiveness of the GBRTM method for real MS event location applications.

#### 3.2.3. Location Results

Location results of the eight blasting events in homogeneous velocity models (use the average value of 3D velocity model) and the high-resolution 3D velocity model using the GBRTM location method are shown in Table 2. The average error of the GBRTM method using the homogeneous velocity model is 39.7 m, while the maximum location error of the GBRTM location method in the 3D velocity model is only 23.0 m and average location error reaches 17.0 m. This again proves the importance of a sufficiently accurate velocity model on the location results of reverse-time migration-based location method utilizing waveform recording.

Fortunately, different ray tracing-based location methods based on arrival time data have been used to locate the above eight blasting events with premeasured locations, which can be used for a quantitative comparison between the GBRTM location method and ray tracing-based location methods. Shang et al. [70] found that there is a very large location error for the blasting event 3 when using the time difference method based on automatic P-wave arrival picking and the average location error of the other events is 91.24 m. Taking the advantages of probability density curve, Dong et al. [71] fitted analytical solutions from different combinations of P-wave arrivals. They took the coordinates with the maximum probability density in each direction as the location result, and their average location error is 47.4 m. Furthermore, Dong et al. [72] removed problematic P-wave arrival data with large picking errors based on the initial location results determined by the above probability density curve, and the average location error of time-difference method using the filtered P-wave arrival data is a bit better (44.6 m) than that without the filtering step, while Li et al. [73] proposed a location method based on virtual fields, with an average location error of 42.0 m. However, the simple homogeneous velocity models used in the above location methods are quite different from the actual complex velocity structure of target mine region. Therefore, Wang et al. [66] located MS events using the ray tracing method in a 3D tomographic velocity model with an average location error of 26.2 m, which illustrates that the location results can be effectively improved by introducing high accurate velocity model. The location result of this study is better than that obtained by the 3D ray tracing method, and there is no need to tediously identify first arrival time nor any dependency on the accuracy of first arrival picking. Furthermore, waveform information considering the finite frequency effects is naturally included in the GBRTM method, facilitating automatic and accurate MS event location.

## 4. Discussions

### 4.1. P-Phase Arrival Picking

The ray tracing-based location method based on travel time heavily relies on the picking accuracy of P-wave arrival. The absolute arrival time picking method and waveform cross-correlation-based relative delay time picking method are usually used to determine P-wave arrival time. However, the amplitude of initial P-wave arrival may be very unclear due to the effect of background noise [23], attenuation of wavefield propagation, scattering, and intrinsic wavefront healing effects [68], which make it hard to reach the required accuracy by using the absolute arrival time picking method. The windowed waveform cross-correlation method mainly depends on the relative delay of the main peak controlled by the dominant period of the target phase waveforms, which greatly reduces the requirements for sharpness of P-wave arrival waveform amplitude. However, when the noise, dispersion, and attenuation effects of propagating wavefields are particularly serious, the main peak distribution of target phase becomes unclear and dispersive, which can result in an obvious picking error when utilizing the cross-correlation picking method. In addition, signal misclassification of adjacent MS events can also lead to large picking error [25]. The above problems make the location results of the ray tracing-based location method based on arrival time picking unstable.

In order to decrease the influences of large picking errors, some researchers take advantage of the points with high probability densities for determining location results of different P-wave arrival subdatasets by clustering analysis and logistics regression. They then chose the mean or maximum location of these points as the final location results [71,72,74]. Nevertheless, locating the source using P-wave arrival subdatasets calls for huge computation. The above methods may fail when small number of P-wave arrivals are available. The location method based on virtual fields proposed by Li et al. [73] reduces contributions of large picking error and remote stations by using an exponential function for objective function on the arrival time difference, but it has to subjectively adjust the weighting factors.

By contrast, for the reverse-time migration and back propagation wavefield-stacking-based Gaussian beam modeling, there is no need to pick P-wave arrival time, which only requires roughly identifying envelopes of initial P-wave arrival. This is very conducive to locating events with low SNR waveforms and automatically identifying MS events. In general, the complex dispersive wave trains (e.g., multiple phases, frequency dispersion or strong scattering) and waveforms with large travel time anomaly are usually recorded by sensors far away from the source. The Gaussian beam wavefield modeling correctly simulating the geometric spreading attenuation could automatically consider and downweight these far-field waveform recordings for the wavefield migration and stacking. Finally, the GBRTM location method utilizes the focusing autocorrelation imaging condition for stacking back propagation wavefields without the need to estimate excitation time of MS event, which reduces the complexity of the problem.

### 4.2. Velocity Model

Velocity model accuracy is closely related to both arrival time ray tracing-based location and migration-based location using waveforms. Due to the difficulty of obtaining a reasonable 3D velocity model in a mining zone, some researchers have to locate MS events using a homogeneous velocity model [73,74]. Other researchers utilized a simplified layered velocity model [75,76] and a sectional homogeneous velocity model in the horizontal direction [1] for source location, showing that the 1D velocity model-based location results are better than those based on the homogeneous velocity model. However, owing to the influences of geological condition, cavity distribution, and mining disturbance, it is still hard to simplify the strong 3D heterogeneous velocity of a mine to 1D model. In view of this, Peng and Wang [77] and Peng et al. [78] adopted a very simple 3D velocity model (homogeneous velocity model including cavities) and traditional ray tracing method for an MS event location, while Wang et al. [66] took advantage of the high-resolution 3D velocity model obtained by travel time tomography [65], making the first implementation of mine MS event location by using 3D velocity model-based ray tracing method in a mine. Their results show that MS event location accuracy based on a high-resolution 3D velocity model is obviously higher than that obtained by the homogeneous velocity models. However, as shown in Figure 1 of Wang’s paper, the ray tracing-based location method has inherent difficulty in multi-path, focusing and defocusing effects modeling for highly heterogeneous media. The Gaussian beam technique used in this study further considers wavefield dynamics information (e.g., changes of wavefield amplitude in heterogeneous media and evolution of wavefront curvature) to correctly model focusing/defocusing and multi-path effects (e.g., Figure 3). The average location error based on the Gaussian beam location method is 17.0 m for the eight real blasting events, which is even better than the results obtained by the 3D ray tracing-based location method using the same 3D model (26.2 m).

The synthetic and application tests both illustrate the importance of an accurate velocity model to the GBRTM location method: using homogeneous velocity model or 1D velocity model usually achieves a location accuracy at the basic level, and a high-resolution 3D velocity model should be adopted if conditions permit. The propagation of wavefields is affected by the velocity anomaly in the entire Fresnel volume around the central ray due to finite bandwidth of wavefield. Compared with the traditional tomography method based on the geometric ray theory, the finite-frequency tomography technique replaces the geometric ray path used in traditional travel time tomography with “wave path” considering the Fresnel volume related to wavefield frequency, which can more accurately characterize the sensitivity of seismic signals to the velocity structure. The “wave path” has the same sensitivity for both the low-velocity and high-velocity region, and reduces the asymmetry problem caused by the sampling difference of the geometric ray path between the high-velocity and low-velocity region.

The velocity model resolution adopted in this study is about 50 m [65], which almost reaches the resolution limit of the traditional ray tracing-based tomography using the available sensor array. The utilization of finite-frequency tomography technique or full waveform inversion (highest resolution but also needs very high computational cost) could further improve the resolution of 3D velocity model, which could obtain a higher location accuracy using the proposed 3D GBRTM location method.

### 4.3. Imaging Condition and Modeling Considerations

The commonly used imaging conditions are the cross-correlation imaging condition [48] and autocorrelation imaging condition [35]. The cross-correlation imaging condition produces a high-resolution imaging and it is easy to pick the energy focusing points, which is suitable for detecting multi-event locations. This type of imaging condition can effectively suppress the incoherent noises in reverse-time migration process. However, it requires back propagating the wavefields using data from each receiver separately before final multiplication operation, and is thus computationally intensive [48]. When inaccurate microseismic waveform records are used for migration, the cross-correlation imaging condition may seriously affect the imaging focusing and location resolution. On the other hand, the autocorrelation imaging condition back-propagates all the windowed waveforms at once, which requires less computation and is more robust due to the stacking nature of the autocorrelation imaging condition [50]. Due to the record collection requirement of simultaneous back propagation operation, the autocorrelation imaging condition is not very suitable for distributed networks. In addition, the systematic noise and error induced by inaccurate velocity model or algorithm imprecision may be amplified through the autocorrelation imaging condition. These imaging artefacts and required wavefield focusing are superposed together, which may reduce the signal-to-noise ratio and resolution of source imaging results [35].Therefore, Sun et al. [48] proposed a cross-correlation and autocorrelation combined imaging condition for the distributed sensor network (DSN) system to consider the advantages of these two types of imaging conditions. It reduces both computation and communication burdens and preserves the spatial location resolution, which makes the high-resolution real-time and in situ microseismic source location possible. After that, Li et al. [50,51] supplemented a high-resolution waveform inversion restricted in a small area of interest to further iteratively improve the location resolution.

In our case, the recorded waveforms have already been separated into event segments by the IMS system in a mine, that is to say, we usually need to locate single event instead of multi-events. Besides, in contrast to general dense deployment of sensor array for microseismic monitoring in exploration of the geophysical field, the limited number of sensors in a mining region are irregularly distributed and the strong heterogeneous media result in incoherent recorded waveforms with rather low similarity. When there are strong random noises and complicated wave train interference in the microseismic records, more sensors are needed to suppress these unwanted noise signals in cross-correlation imaging condition, which will increase the corresponding computation burdens. Considering the demand of location result robustness, it may be problematic to implement the cross-correlation imaging condition for strong heterogeneous mine velocity model and a small number of underground mining sensors. 

Therefore, the imaging condition used in our study is zero-lag of the autocorrelation at every spatial location [35]. Compared with other stacking types of imaging condition, such as the maximum or average amplitudes of waveform recording over time [53,55], utilizing autocorrelation operation (proportional to the variance for zero-mean data) collapses complicated waveforms and captures the total energy of microseismic event in the data domain. It is also especially beneficial when the waveform records suffer from poor signal-to-noise ratio, which is the case in this study. In the future, with the increase of the mine monitoring sensor density and the improvement of the algorithm, a more sophisticated hybrid correlation-based imaging condition will be developed to further improve the resolution [49,59].

The simplified Gaussian beam construction [59] is very suitable for real-time MS event location, which is based on the assumption that only the wave propagation paths from receivers to sources need to be considered. However, there are multi-ray path effects as well as waveform focusing and defocusing in mining engineering and, thus, we used the traditional Gaussian beam technique for more stable inversion. In fact, we can use the reciprocity theorem to pre-compute the wavefield library from sensors to each element, which also makes real-time MS event location possible.

Compared to our standard Gaussian beam migration technique, the simplified Gaussian beam construction proposed in [59] is very suitable for in situ and real-time MS event location, which is based on the assumption that only the wave propagation paths from receivers to sources are considered by beamforming technique, such that the corresponding simplified Gaussian beam time reversal imaging (SGTRI) method only calculates the backward-propagated wavefield in the range of one single Gaussian beam, which is only one-tenth or even less of the computational cost of the whole wavefield extrapolation. However, complex multi-path propagation as well as waveform focusing and defocusing effects may occur under complicated field conditions for mining engineering and, thus, we decided to use the standard Gaussian beam modeling technique based on complete 3D ray tracing for more stable source inversion. In fact, we can pre-compute the travel time and ray path table from each sensor to candidate spatial grid, which also makes the real-time MS event location by our GBRTM reasonably efficient [51].

## 5. Conclusions

This research introduced the GBRTM technique into a mine MS event location and considered the high-resolution 3D velocity model for wavefield back propagating. The synthetic test shows that the spectrum distribution characteristics of the realistic blasting waveforms are more complicated than the synthetic waveforms excited by the popular Ricker source wavelet and, thus, using synthesized waveforms based on recorded waveforms can provide a more reliable way to verify the applicability and robustness of a location method. Wavefield back propagation based on the Gaussian beam modeling considers the finite frequency wavefront healing effects in a complex velocity model, which is more accurate than traditional ray tracing modeling. In addition, the GBRTM technique directly utilizes the windowed waveforms containing initial P-wave arrival to locate MS events through wavefield back propagation and stacking, without demanding manual or unstable automatic picking of P-wave arrival. Therefore, it is very suitable for automatic event location with low SNR waveforms, and has good resistance to large P-wave arrival picking errors. The synthetic and application tests prove that the location accuracy of the GBRTM location method in a 3D velocity model is obviously higher than that in a homogeneous or simple 1D model, which indicates that velocity model is crucial to reverse-time migration-based location methods. The average location error of the GBRTM location method is just 17.0 m for the eight blasting events, which is better than that of the ray tracing-based location method when using a homogeneous velocity model (>40 m) and the same 3D velocity model (26.2 m). If the finite frequency effect modeling is considered to further improve the resolution of the 3D velocity model in a mine region, the GBRTM location method is expected to achieve better location accuracy. Due its location effectiveness, we will embed the GBRTM location method into our RHMS (real-time, high-precision, and multi-scale MS monitoring system) software and release it in the future.

## Figures and Tables

**Table 1:** Location errors (m) of the 15 test events based on the GBRTM location method using the correct 2D velocity model shown in Figure 2.

Test Event id	1	2	3	4	5	6	7	8	9	10	11	12	13	14	15
Location error (m)	0.4	1.3	2.7	2.4	2.9	2.0	0.1	2.5	2.8	2.0	2.3	2.2	1.2	2.0	0.5

**Table 2:** Location errors (m) of the eight blasting events based on the GBRTM location method using the 3D velocity model and homogeneous velocity model.

Blasting Event ID	1	2	3	4	5	6	7	8
**Homogeneous velocity-based location error (m)**	44.4	36.2	31.6	27.3	36.6	37.8	34.9	68.8
**3D velocity model-based location error (m)**	23.0	21.1	5.1	24.2	7.2	23.8	18.2	13.8

## Supplementary Materials

The following are available online at https://www.mdpi.com/1424-8220/20/9/2676/s1.
