# Supplementary material for: Locating Mine Microseismic Events in a 3D Velocity Model through the Gaussian Beam Reverse-Time Migration Technique
Source: Sensors (Basel). 2020 May 8;20(9):2676. doi: 10.3390/s20092676 (PMC7248888; doi:10.3390/s20092676)
Supplement: Supplementary file 1 [file sensors-20-02676-s001.pdf]

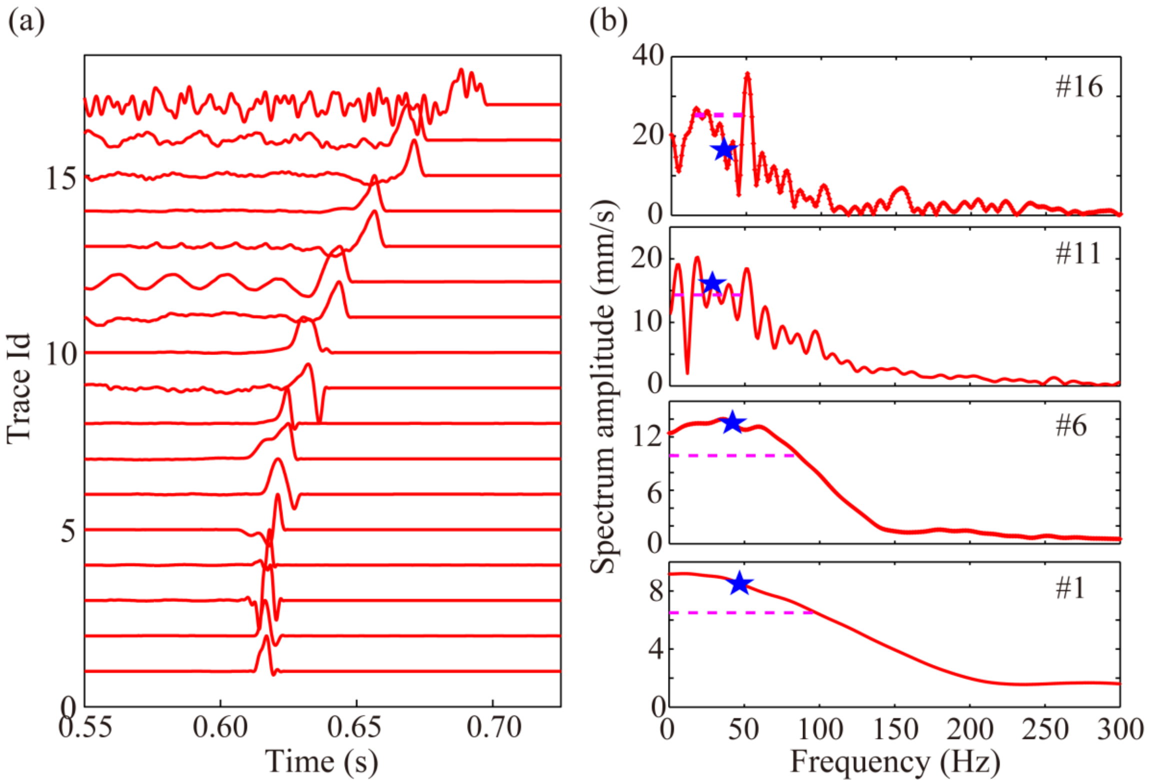

Figure S1. Windowed waveforms of the realistic blasting event 3 (a) and representative amplitude spectra of some windowed waveforms (b). The blue star represents the main frequency of the waveform and the pink dashed line corresponds to the approximate dominant frequency band.

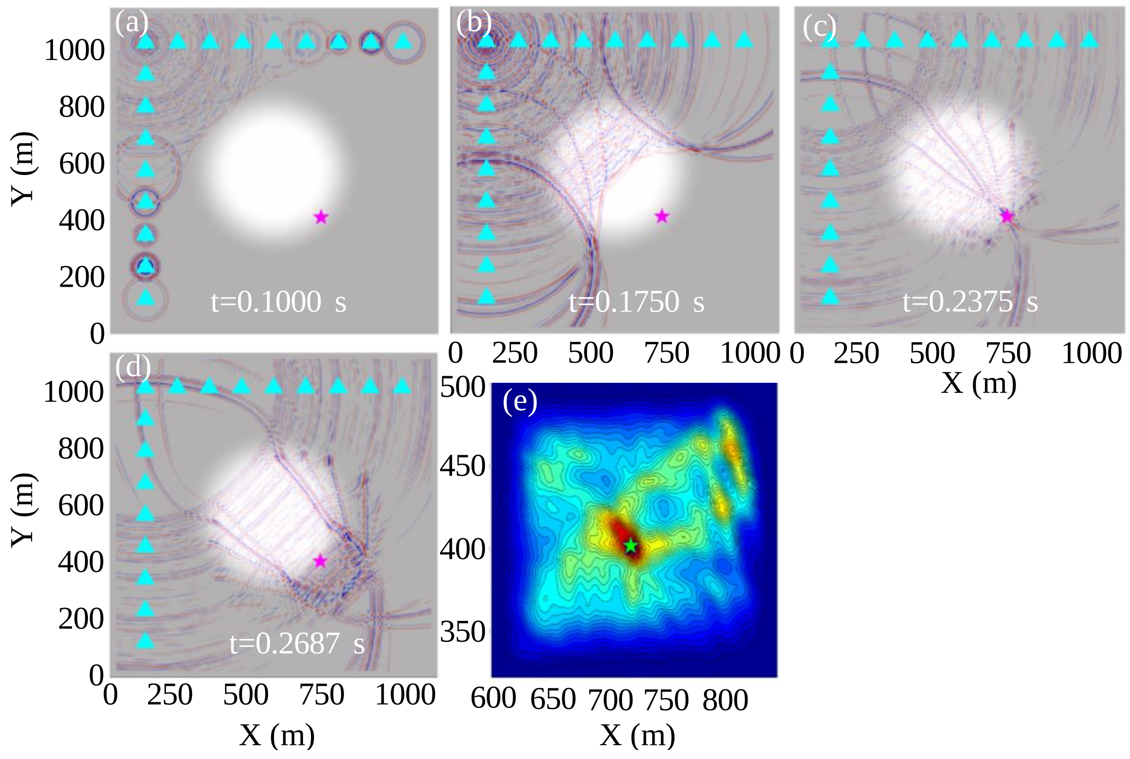

Figure S2. Location results of the GBRTM technique when using synthetic waveforms from the realistic blasting event 3. The rest of the instructions are the same as in Figure 3.
